# Supplementary material for: WNT4 Regulates Cellular Metabolism via Intracellular Activity at the Mitochondria in Breast and Gynecologic Cancers
Source: Cancer Res Commun. 2024 Jan 17;4(1):134–51. doi: 10.1158/2767-9764.CRC-23-0275 (PMC10793200; doi:10.1158/2767-9764.CRC-23-0275)
Supplement: Supplemental Figure 1 — BioID setup and proof-of-concept [file crc-23-0275-s01.pdf]

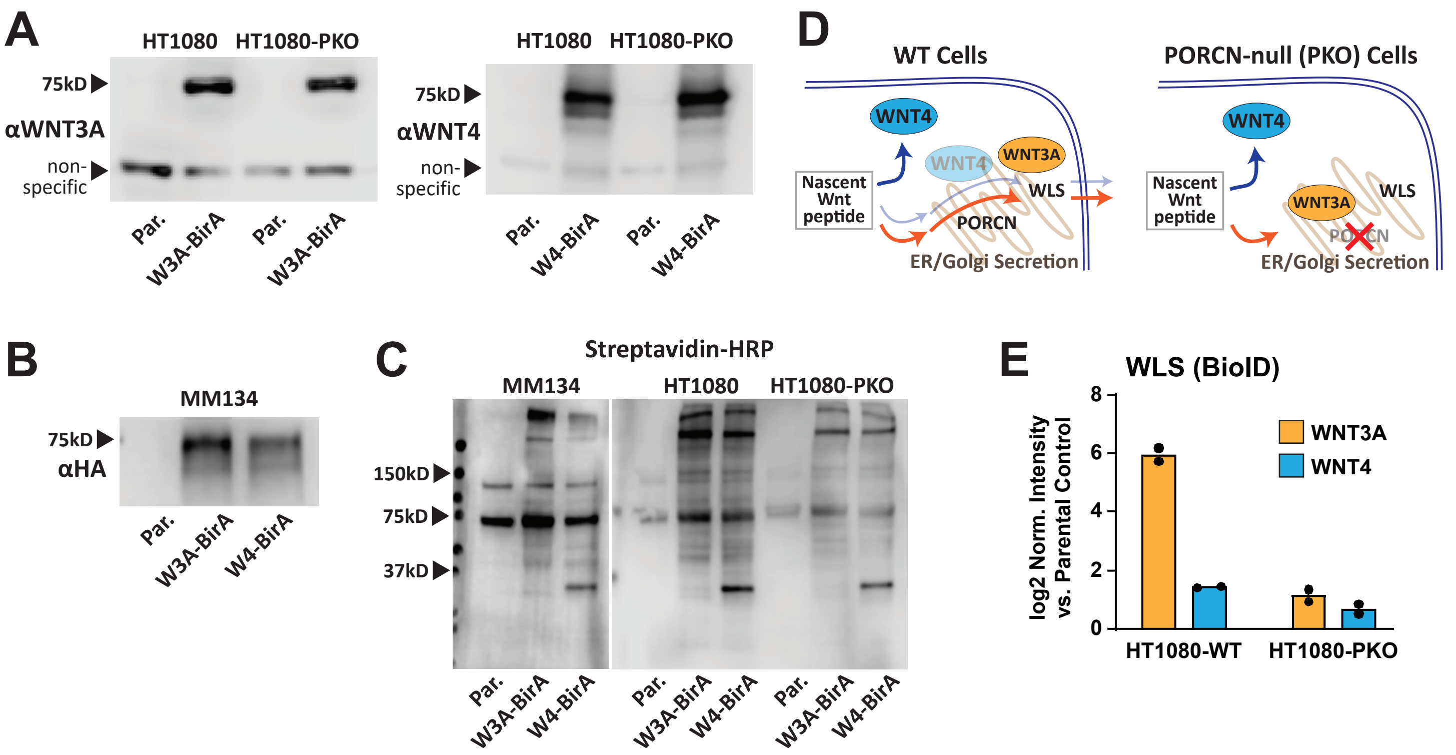

Supplemental Figure 1. Wnt-BirA proximity biotinylation system differentiates PORCN-mediated Wnt trafficking.
(A-C) Immunoblotting of Parental cell lines (Par.) or cells stably expressing the indicated Wnt-BirA fusion protein. **(A)** Wnt-BirA fusions are predicted at ~75kD, and are detected by WNT3A or WNT4 antibodies at the expected size. Non-specific band shown as loading confirmation. **(B)** Wnt-BirA fusions can also be detected at the expected size using an internal HA-tag. **(C)** Parental and Wnt-BirA expressing cells were treated with 50μM biotin for 24hr, and whole cell lysates were analyzed for the presence of protein biotinylation using streptavidin-HRP conjugate. Wnt-BirA expressing show a broad range of protein biotinylation not observed in parental cells. **(D)** Model of WNT3A vs WNT4 trafficking in PORCN-wild type vs -knockout contexts. WNT4 trafficking to its intracellular localization is to be PORCN-independent, while WNT3A trafficking to the ER/Golgi and secretion is PORCN-dependent. **(E)** Mass spectrometry intensity for WLS in the indicated Wnt-BirA model versus parental cell control. Points represent biological duplicate samples.
