# Supplementary material for: WNT4 Regulates Cellular Metabolism via Intracellular Activity at the Mitochondria in Breast and Gynecologic Cancers
Source: Cancer Res Commun. 2024 Jan 17;4(1):134–51. doi: 10.1158/2767-9764.CRC-23-0275 (PMC10793200; doi:10.1158/2767-9764.CRC-23-0275)
Supplement: Supplemental Figure 2 — RPPA dataset clustering [file crc-23-0275-s02.pdf]

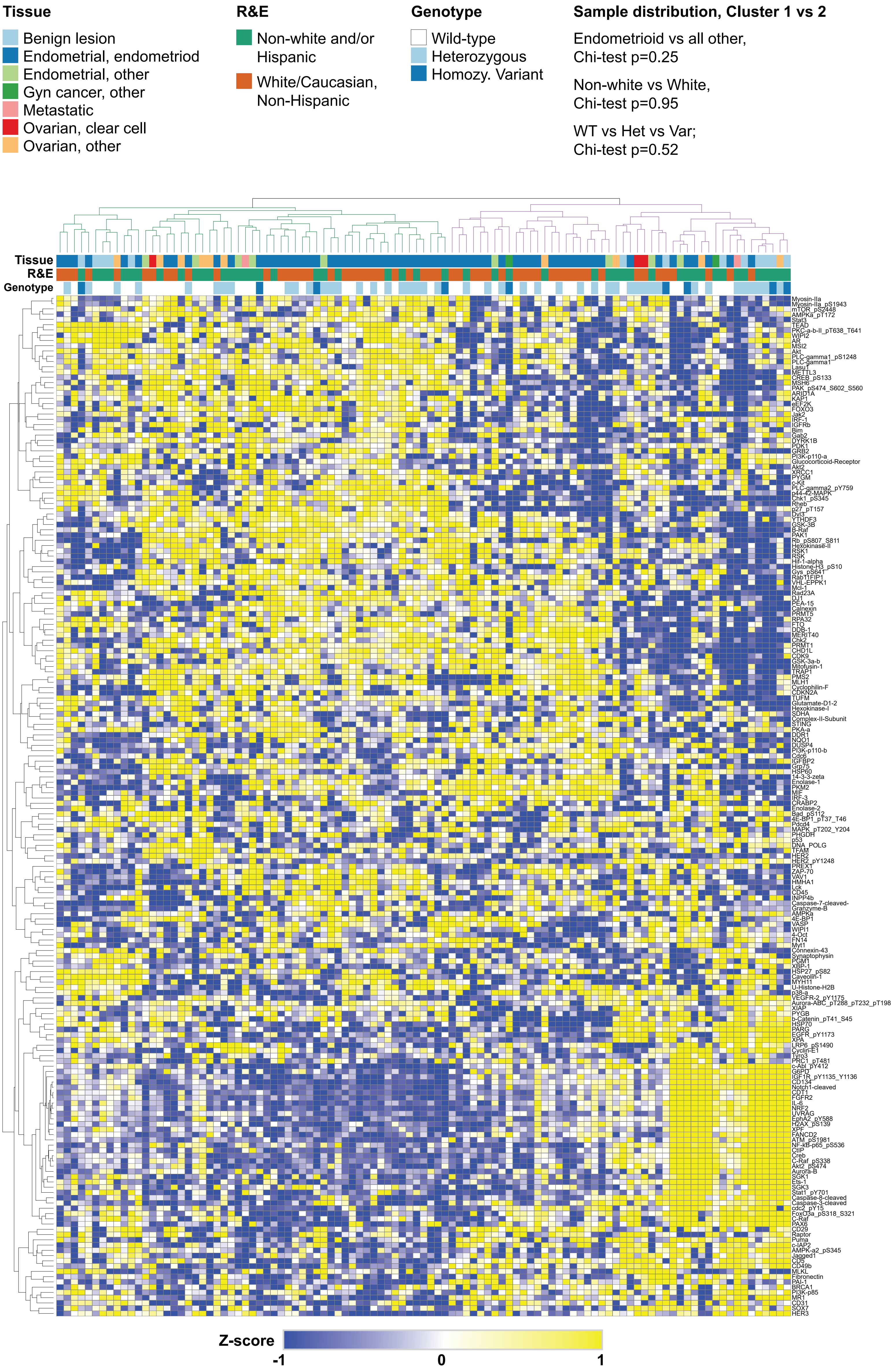
Tissue
Benign lesion
Endometrial, endometriod
Endometrial, other
Gyn cancer, other
Metastatic
Ovarian, clear cell
Ovarian, other
R&E
Non-white and/or Hispanic
White/Caucasian, Non-Hispanic
Genotype
Wild-type
Heterozygous
Homozy. Variant
Sample distribution, Cluster 1 vs 2
Endometrioid vs all other, Chi-test p=0.25
Non-white vs White, Chi-test p=0.95
WT vs Het vs Var; Chi-test p=0.52
Tissue
R&E
Genotype
Myosin-IIa
Myosin-IIa_pS1943
mTOR_pS2448
AMPKa_pT172
Stat3
TEAD
PKC-a-b-II_pT638_T641
WIPI2
AR
MSI2
Akt
PLC-gamma1_pS1248
PLC-gamma1
Lasu1
METTL3
CREB_pS133
MSH6
PAK_pS474_S602_S560
ARID1A
KAP1
eEF2K
FOXO3
Jak2
IRF-1
IGFRb
Bim
Gab2
DYRK1B
PDK1
GRB2
PI3K-p110-a
Glucocorticoid-Receptor
Akt2
XRCC1
PYGM
c-Kit
PLC-gamma2_pY759
p44-42-MAPK
Chk1_pS345
Rheb
p27_pT157
Dvl3
YTHDF3
GSK-3B
B-Raf
PAK1
Rb_pS807_S811
Hexokinase-II
RSK1
RSK
Hif-1-alpha
Histone-H3_pS10
Gys_pS641
Rab11FIP1
VHL-EPPK1
Mcl-1
Rad23A
DJ1
PEA-15
Calnexin
PRMT5
RPA32
FTO
DDB-1
MERIT40
Chk2
PRMT1
CHD1L
CDK9
GSK-3a-b
Mitofusin-1
TRAP1
PMS2
MLH1
Cyclophilin-F
CDKN2A
TUFM
Glutamate-D1-2
Hexokinase-I
SDHA
Complex-II-Subunit
STING
PKA-a
DDR1
NQO1
DUSP4
PI3K-p110-b
Cdc6
IGFBP2
Grp75
HSP60
14-3-3-zeta
Enolase-1
PKM2
MIF
IRF-3
CRABP2
Enolase-2
Bad_pS112
4E-BP1_pT37_T46
Pdcd4
MAPK_pT202_Y204
PHGDH
p53
DNA_POLG
TFAM
HER2
HER2_pY1248
PREX1
ZAP-70
VAV1
HMHA1
Lck
CD45
INPP4b
Caspase-7-cleaved-
Granzyme-B
AMPKa
4E-BP1
VASP
WIPI1
4-Oct
FN14
Myt1
Connexin-43
Synaptophysin
PGM1
XBP-1
HSP27_pS82
Caveolin-1
MYH11
U-Histone-H2B
p38-a
VEGFR-2_pY1175
Aurora-ABC_pT288_pT232_pT198
XIAP
PYGB
b-Catenin_pT41_S45
HSP70
PARG
EGFR_pY1173
XPA
LRP6_pS1490
Cyclin-E1
Tyro3
PRC1_pT481
c-Abl_pY412
G6PD
IGF1R_pY1135_Y1136
CD134
Notch1-cleaved
CDT1
FGFR2
IL-6
NRF2
UVRAG
EphA2_pY588
H2AX_pS139
XPF
FANCD2
ATM_pS1981
NF-kB-p65_pS536
CtIP
Creb
C-Raf_pS338
Akt2_pS474
Aurora-B
SGK1
Ets-1
SGK3
Stat1_pY701
Caspase-8-cleaved
Caspase-3-cleaved
cdc2_pY15
FoxO3a_pS318_S321
C-Raf
PAX6
CD29
Raptor
Puma
c-IAP2
AMPK-a2_pS345
Jagged1
CD5
CD49b
MLKL
Fibronectin
PAI-1
BRCA1
PI3K-p85
MR1
CD31
SOX7
HER3
Z-score
-1
0
1

Supplemental Figure 2. Differential protein array signals across gynecologic tissue cohort.
Top protein array targets based on differential levels in wild-type vs variant genotype tissues were subject to hierarchical clustering (Pearson) using Morpheus. Two major sample clusters (Green vs Purple tree outlines at top) were not significantly different regarding distribution of tissue type, race & ethnicity, and rs3820282 genotype.
