# Supplementary material for: WNT4 Regulates Cellular Metabolism via Intracellular Activity at the Mitochondria in Breast and Gynecologic Cancers
Source: Cancer Res Commun. 2024 Jan 17;4(1):134–51. doi: 10.1158/2767-9764.CRC-23-0275 (PMC10793200; doi:10.1158/2767-9764.CRC-23-0275)
Supplement: Supplemental Figure 3 — RPPA networks in full cohort dataset [file crc-23-0275-s03.pdf]

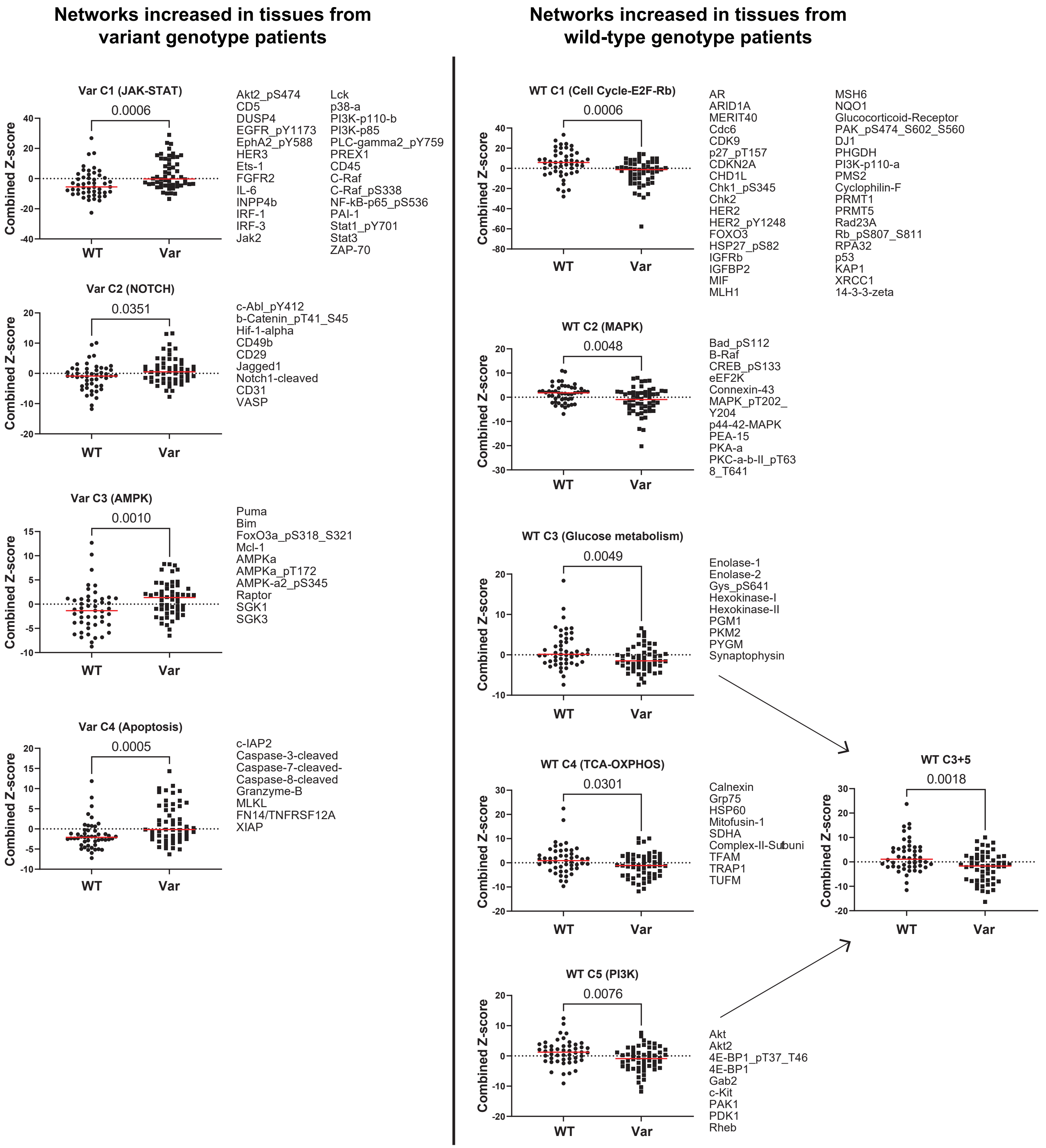

Supplemental Figure 3. Protein signaling networks show differential activity by rs3820282 genotype. STRING networks defined by MCL clustering; networks shown with >6 protein array targets included (also see Figure 6). Points represent individual tissue samples and the sum of z-scores for the listed array targets. Red line = median score; comparisons by Mann-Whitney T-test.
